# Analysis of CEPH-accredited online public health doctoral programs and MPH degree holder demand

**DOI:** 10.3389/fpubh.2023.1053531

**Published:** 2023-05-25

**Authors:** Barbara Lewis, Francisco Sy

**Affiliations:** ^1^Department of Health Care Administration, School of Public Health, University of Nevada, Las Vegas, Las Vegas, NV, United States; ^2^Department of Environmental and Occupational Health, School of Public Health, University of Nevada, Las Vegas, Las Vegas, NV, United States

**Keywords:** online, doctorate, public health, DrPH, CEPH, MPH, doctoral program

## Abstract

**Introduction:**

The number of doctoral programs to train future public health leaders is critical in meeting the demand of global health challenges in the 21st century. Ten United States online public health doctoral programs accept only a fraction of interested learners.

**Methods:**

This research examines the launch of the first online public health doctoral program, accredited by the Council on Education for Public Health, and compares nine similar programs that have followed in the ensuing 12 years.

**Results:**

Survey results highlight the demand by Master of Public Health degree holders for online public health doctoral programs; 84.11% of survey respondents indicated an interest in obtaining a doctoral degree.

**Discussion:**

If we strive to answer the question posed by the Institute of Medicine in 2003, “Who will keep the public healthy?” then we need to provide education that is accessible, efficient and equitable for interested learners, most of whom are turned down by online public health doctoral programs that have limited capacity.

## Introduction

Prior to the COVID-19 pandemic in 2020, there were six online doctoral programs in public health accredited by the Council on Education for Public Health (CEPH). As of January 2022, four additional programs had joined the ranks, responding to the health crisis challenging countries around the globe by allowing eligible students from anywhere in the world to obtain a doctoral degree in public health.

These institutions answered the question posed by the Institute of Medicine (IOM), now known as the National Academy of Medicine, which released a seminal report in 2003, “Who Will Keep the Public Healthy?” (1). The report recommended that graduate trainee programs: (1) train all graduates in the ecological model of health determinants; (2) train all graduates in eight new content areas: informatics, genomics, communication, cultural competence, community-based participatory research, global health, policy and law, and ethics; (3) expand supervised practice opportunities as part of the formal core curriculum; and (4) increase collaboration with policy makers, practitioners, communities, and other academic disciplines.

In their journal commentary, Schlaff and colleagues point out the difficulty in combining disparate content areas integrated in one program (2). Indeed, none of the 10 online public health doctoral programs in this study incorporated all of the IOM recommended content areas. The authors suggest teaching the ecological model (3) and including a practice model in advanced degree programs.

To address the IOM recommendations, the Association of Schools and Programs of Public Health (ASPPH), a membership organization of CEPH-accredited public health schools and programs, developed DrPH Foundational Competencies, which were identified as a national consensus in 2009 (4). These competencies are advocacy, communication, community/cultural orientation, critical analysis, leadership, management, and professionalism and ethics.

The DrPH degree got its start in 1919 under the direction of the American Public Health Association. The Committee of Sixteen issued a report on the Standardization of Public Health Training because of the variety of previous training and degree requirements across 16 United States schools. The committee recognized the DrPH degree as advanced education for graduates in medicine and included practical field work (5). The PhD, on the other hand, is considered an academic degree (6).

The first school to implement a synchronous online doctoral program in public health was the University of North Carolina at Chapel Hill Gillings School of Global Public Health (UNC) in 2005. Suspending their residential program, which was created in response to the IOM report, UNC reengineered the program for delivery via distance to better serve the needs of professionals working full-time in field positions, the target audience for the program. This modality ensured equitable access for professionals who were not resident in the Chapel Hill area of North Carolina so they could attend classes in person.

An advisory group created a new competency model for the reengineered curriculum, and Suzanne Babich, DrPH, MS, who graduated from the residential program in 2001 and taught full-time in the UNC Department of Health Policy and Management, worked with public health faculty member Ned Brooks, DrPH. He was the founding program director who had recently come to the school of public health after working at UNC’s Office of the Provost. The curriculum was updated and designed to emphasize highly interactive, small group discussions and debates to provide the experiential learning required for a program focused on building leadership skills. Like the in-person DrPH program, the degree targeted diverse professionals with practice-oriented career goals in contrast to PhD programs, which prepare recipients for academic research and teaching.

The advisory group decided on a health leadership degree with only small cohorts of 10 to 12 learners. Babich explained the rationale for why they wanted a small cohort. “You cannot teach leadership didactically; the emphasis needs to be on experiential learning” (S. Babich, personal communication, 9 September 2022). Within 1 year, the online program was ready to launch as the first synchronous internet video based doctoral program in public health (7). The program started in 2005, about 1 year after the proposal was sent through the UNC system for approval, with an initial cohort of nine, all women. The acceptance rate for the first several years held steady at about 8% to10% of between 80 to130 applicants. That number of applicants ballooned to more than 200 during the pandemic when interest in public health programs increased (S. Babich, Personal communication, 9 September 2022). Originally, the focus was on domestic students, but in 2007 the program tested and then began to admit international students, adding cultural and geographic diversity.

After every semester, Babich conducted focus groups giving students the power to suggest changes. The program underwent an extensive evaluation after several years of operation. Babich identified three keys to success that she described as the “secret sauce” (S. Babich, personal communication, 19 January 2022):

1. Each cohort moves together in lockstep. It does not matter if someone is a world expert in a topic, everyone takes every course, with cohort members learning from the inputs of others in their cohort.
2. Synchronicity—Real-time classes with the ability to have highly interactive discussions was vital. Concerns about challenges due to time zone differences never materialized.
3. Face-to-face residential sessions—three times a year in each of years one and two of the program. Participants usually meet at the Chapel Hill campus, but the program occasionally meets overseas (face-to-face residential sessions were temporarily paused during the COVID-19 pandemic).

Initially, marketing was by word of mouth only. With UNC’s 14-year history of a residential DrPH program, coupled with the low number of people who could be admitted, there were plenty of applicants. UNC maintained high retention and completion rates. On the rare occasion when someone would take a leave of absence or abandon the program, it was generally spurred by personal challenges. These issues, for example, included stressful life events such as divorce, death of a family member, sickness and, occasionally, a job change.

The UNC DrPH program began to break even financially in year 3. Eighty percent of the budget was for program faculty which numbered approximately 15. Babich estimates that expenses were about $800,000 a year (S. Babich, personal communication, 19 January 2022).

In 2010, Loma Linda University School of Public Health (LLU) and the University of Illinois at Chicago School of Public Health (UIC) launched the second and third online doctoral programs in public health—both CEPH accredited. Five years later, at the behest of her former colleague, Indiana University Richard M. Fairbanks School of Public Health (IU) Founding Dean, Paul Halverson, Babich moved to IU and launched the “2.0 version” of the UNC program, updated with a globalized curriculum, a number of international faculty and substantial collaboration with international partner universities in Kenya and the Netherlands (S. Babich, personal communication, 9 September 2022). IU’s DrPH is described as a Doctoral Program in Global Health Leadership. It launched in 2018 and admits two cohorts each year of approximately 12 to 15 mid to senior level and junior level participants.

Joshi and Amadi conducted a study of 85 CEPH-accredited public health programs offering various degrees and certificates in 2014 (8). However, the purpose of this research was to narrow the focus to only CEPH-accredited online public health doctoral programs and add a critical dimension with the inclusion of survey results from potential candidates who may be interested in enrolling in those programs. To our knowledge, this is the first study that compares online public health doctoral programs and survey results from potential learners about their interest in online degree programs.

## Methods

### Institutional information

Using a mixed methods research design, information about the online public health doctoral programs was gathered in four ways: literature review, internet search, emails to school contacts and conversations with those contacts. In early 2022, we conducted a literature review using the key words DrPH, PhD, online and remote, and public health in the PubMed database. Only nine articles were relevant to this research. We also searched Google and Google Scholar. We included the years from when the first online DrPH program launched in 2005.

The internet research on CEPH-accredited online public health doctoral programs revealed two websites that aggregate college data including www.thebestschools.org and www.bestcolleges.com. After following the links to the schools’ websites, we discovered that much of the data was inaccurate. Ten colleges did not have online public health doctoral programs; however, 15 institutions did.

The next step was to cross reference the 15 institutions to identify which schools had online programs accredited by CEPH, which was established in 1974 as an independent agency recognized by the United States Department of Education to accredit public health schools and programs. The ASPPH website lists CEPH-accredited schools and programs in public health. Of the 15 institutions, five were not accredited by CEPH, including Capella University, the University of Phoenix, and Walden University, which were accredited by the Higher Learning Commission; Northcentral University accredited by the Accrediting Commission for Schools Western Association of Schools and Colleges; and Samford University, which was accredited by the Southern Association of Colleges and Schools Commission on Colleges.

The following is a list of the public health schools and programs (and year the program launched). These programs offer CEPH-accredited online doctoral degrees, all of which are DrPHs. University of North Carolina Gillings School of Global Public Health (2005), Loma Linda University School of Public Health (2010), University of Illinois at Chicago School of Public Health (2010), University of South Florida College of Public Health (2014), Johns Hopkins Bloomberg School of Public Health (2016), Indiana University Richard M. Fairbanks School of Public Health (2018), Georgia Southern University Jiann-Ping Hsu College of Public Health (2020), Rutgers School of Public Health (2020), University of Nebraska Medical Center College of Public Health (2021), and Mercer University Department of Public Health (2022).

Accessing the schools’ websites aided in populating the institutional information form developed to help gather relevant data. Most of the data were available. For missing data, we reached out to school contacts, who were listed on the website for each school and sent emails that included unanswered questions. Only four institutions of 10 responded to the first email in January 2022. After each response, an online meeting was requested to ask more sensitive questions, such as the number of applicants, the marketing techniques, etc. A second email was sent out 2 weeks later to the schools that did not respond to the first email. All institutional contacts replied by the end of February 2022.

### Potential learners’ survey

The second phase of the research was to survey potential public health doctoral applicants to uncover their perspectives and knowledge, plus understand the demand for online DrPH public health programs. Since nearly every school in the study required an MPH, those degree holders were the target. According to ASPPH, the number of MPH graduates from 2010 to 2021 was 94,470. The survey was customized from a validated survey developed by Madison and colleagues (9). In May and June 2022, we contacted10 organizations about distributing the Qualtrics survey link to their constituents. We contacted MPH and public health groups on LinkedIn and Facebook, as well.

## Results—institutions

Table 1 provides an overview of the 10 CEPH-accredited DrPH programs with the explanations below. All CEPH-accredited online public health programs offer DrPH degrees for individuals, who have been working for 3 to 5 years. IU requires 5 years of management experience for their senior level program. The prerequisites include a master’s degree, usually a Master of Public Health, or core courses. One school requires 3 to 5 years of work for their two programs—epidemiology and emergency preparedness, but no advanced degree. Two institutions require a master’s degree from an accredited school—one from a CEPH-accredited school.

**Table 1:** Overview of the 10 CEPH-accredited online DrPH programs.

	Prerequisite degree	Work requirement	Credits		Syn/Asyn	Cohort	Students	Duration		Faculty	Emersion	
			Grad	Transfer		#	#	Years	Limit	#	Days	#
1	MPH or core courses	None listed	60	9	A	1	8–10	3–4	7	37	virt	
2	Masters or doctoral	PH work, 5 years mgmt	45		S	2	10–15/25	3	3		2–3	3
3	MPH or similar	3 years public health	64		S/A	3	90–100	4–9	9	180	7	2
4	MPH and behavior health course	None listed	62–65		S/A	1	10–12	3	5	7		
5	MPH from CEPH accredited	None listed	57	6	S	2	26	7 sem	5	5 F/T	virt	
6	Master’s degree	5 years work experience	48		S	2	22	4	8.5		2–3	7–8
7	Masters from accredited school	3 years public health	96 h	32 h	S	1	20–25	4–6	7	13	3	1
8	Graduate degree	5 years of work	45–51		S	1	15	3–5	8	20	4–5	6
9	No master’s degree required	3 years public health	54		A	1	17	3	5	3:1		
10	Master’s degree	None listed	43	12	A	2	20–30	3–4	8	10	5	3

### Credits and transfers

Required credits for graduation range from 43 to 65 with several institutions allowing transfer credits.

### Asynchronous vs. synchronous

The institutions are split on asynchronous vs. synchronous with five schools offering synchronous learning, three asynchronous and two schools fielding a hybrid with both synchronous and asynchronous courses.

### Number of students

Half of the schools have one cohort with between 10 to 17 students. IU has two cohorts segmented by years of work and others have cohort sections by tracks. All institutions saw an increase in applications when the COVID-19 pandemic arrived. The ratio of applicants to acceptances ranges from 1 acceptance to 5 applicants at one school to 1 acceptance to 12 applicants at another.

### Faculty

Faculty numbers range from 5 full-time to 180 at one school with 90 to 100 students. Ratios range from two to five students to one faculty member.

### Duration

The duration of the programs ranges from 3 to 9 years with a limit of up to 9 years. The average duration for synchronous programs, where students attend in lockstep, is 4 years.

### Immersion program

Most programs have an in-person immersion at least once a year for a few days to a week. Two institutions hold virtual orientations.

### Costs

Unit costs vary across the programs, including cost per credit, per course and per semester. In some cases, costs differed between in state and out-of-state students. The per credit costs for in-state students range from a low of $277 to $1,233 with an average amount of $769 per credit. Out of state student costs range from $277 to $1,500 per credit. Five of the 10 schools do not charge a different amount between in state and out-of-state students.

### Tracks

The 10 schools offer 11 tracks, with one school offering five tracks as listed in Table 2. Seven of the 10 schools offer a leadership track, although known by different names (e.g., Global Leadership, Health Leadership, etc.). Schools that launched in the early years of DrPH programs included leadership tracks, whereas more recent degree programs have included more diverse tracks based on demand.

**Table 2:** Tracks offered by the 10 institutions.

	Tracks	1	2	3	4	5	6	7	8	9	10	Total
1	Clinical Laboratory Science and Practice										1	1
2	Diverse Populations and Health Equity					1						1
3	Emergency preparedness								1			1
4	Environmental Health			1								1
5	Epidemiology								1			1
6	Health Education				1							1
7	Health Equity and Social Justice			1		1						2
8	Health Policy and Management			1								1
9	Implementation Science			1								1
10	Leadership	1	1		1		1	1		1	1	7
11	Policy and Evaluation			1								1
	Total	1	1	5	2	2	1	1	2	1	2	18

### Marketing

Applicants were recruited through several sources including word of mouth, information sessions, alumni associations, faculty, internet keyword purchase, and social media. Schools frequently used two organizations for their marketing. One was the Schools of Public Health Application Service (SOPHAS), which is a centralized application program where students can submit one application that is distributed to institutions which they select. The link connects to the ASPPH Program Finder where schools’ degrees are listed. The second organization is This Is Public Health, which hosts virtual and in-person events for individuals interested in pursuing degrees. A Graduate School Fair, held virtually on 19 January 2022, had 60 institutions participating. Seven of the 10 schools in this study attended the event. The schools paid a fee of $275 for early registration and $350 for late registration.

## Results—survey

Of 431 survey respondents, 107 held MPH degrees. Though we originally powered the analysis at 95% confidence level with a 5% margin of error, we obtained fewer MPH degree holders than anticipated; hence, our margin of error for MPH holders was 8%. However, the total number of surveys was 431, and hence we provide results separately for MPH and non-MPH degree holders along with overall responses of the combined groups.

The introduction explained the survey to respondents and was the basis for the first several survey questions: “We are conducting an anonymous national survey to understand the interest in online public health doctoral degrees from schools accredited by the Council on Education for Public Health (CEPH). This organization accredits schools such as the Johns Hopkins Bloomberg School of Public Health and the University of North Carolina Gillings School of Global Public Health. Doctoral degrees include the Doctor of Philosophy (PhD), which is usually focused on research, most often in academia; and the Doctor of Public Health (DrPH), which is designed for practitioners who wish to work in public health leadership positions in the field. This survey is about your interest in an online (synchronous with learners in a virtual class together) DrPH degree, which is for people who are working and takes 3 to 4 years to complete. Your dissertation would be a project within your organization.”

Here are the highlights from MPH degree holders, non-MPH degree holders and both aggregated as shown in Table 3. Interestingly, about 12% of respondents indicated that they had not heard of a DrPH degree before they read the survey introduction, but that number jumped to over half of non-MPH degree holders. Despite the fact that only 12% of MPH degree holders had not heard of DrPH degrees, over 30% did not know the difference between a DrPH and PhD compared to nearly 70% of non-MPH degree holders.

**Table 3:** Delineates the difference in responses among MPH degree holders, non-MPHs and all respondents.

Question	Choice	MPH	Non-MPH	All
Before you read the description above, had you heard of the DrPH degree?	Yes	87.85%	45.96%	56.48%
	No	12.15%	54.04%	43.52%
Before you read the description above, did you know the difference between PhD and DrPH degrees?	Yes	69.81%	30.22%	40.23%
	No	30.19%	69.78%	60.00%
Do you believe that a DrPH degree could advance your career?	Definitely yes	23.08%	24.30%	23.67%
	Probably yes	41.35%	32.71%	34.80%
	Might or might not	23.08%	23.99%	23.43%
	Probably not	12.50%	13.40%	13.23%
	Definitely not	2.88%	5.61%	4.87%
Have you ever thought about going back to school to get a doctoral degree?	Yes	84.11%	60.25%	66.51%
	No	11.21%	33.12%	27.40%
	N/A	4.67%	6.62%	6.09%
What has prevented you from doing so? (check all that apply.)	#1 Reason cost	73.83%	67.71%	32.46%
	#2 Reason—time	63.55%	55.80%	26.80%
Does your employer provide tuition reimbursement?	Yes	35.51%	33.85%	34.03%
	No	42.06%	30.75%	34.03%
	Do not know	16.82%	31.06%	27.31%
	I am not working	5.61%	4.35%	4.63%
Would you be interested in learning more about an online DrPH degree from a prestigious school?	Yes	64.49%	36.99%	44.29%
	Maybe	18.69%	29.15%	26.34%
	No	16.82%	33.86%	29.37%

Noteworthy is that nearly two thirds of MPH degree holders indicated that a DrPH degree could definitely or probably advance their careers. Both MPH and non-MPH degree holders overwhelmingly have thought about pursuing a doctoral degree. Both cite cost as the primary barrier, yet over one third of non-MPH degree holders do not know if their employers provide tuition reimbursement and about half as many MPH degree holders indicated the same.

Of the seven DrPH foundational competencies developed by the ASPPH, MPH respondents were most interested in leadership at 69.81% followed by management (60.38%), critical analysis (58.48%), advocacy (54.72%), community/cultural orientation (48.11%), communication (36.68%), and professionalism and ethics (35.85%).

However, when the 11 tracks in current online public health doctoral programs were listed, over half of the respondents selected epidemiology as a potential focus (54.81%,), followed by diverse populations and health equity (47.12%). There was almost equal interest (36%–38%) in health policy management, health equity and social justice, policy and evaluation, and leadership. In the bottom segment were health education (29.81%,), environmental health (25.00%), emergency preparedness (23.08%), implementation science (18.72%), and clinical laboratory science and practice (10.58%).

## Discussion

Although the number of CEPH-accredited online public health doctoral programs doubled in the last 5 years, the growth has not kept pace with the demand. Like other health professions programs, the number of applicants has exploded during the past 2 years, which may be due to the interest in epidemiology and health equity as evidenced by the survey results. In the 10 programs studied, the number of applicants is, on average, more than seven times the number of acceptances. The low number of programs, coupled with the small cohorts of only 10 to 17 students on average, could be an issue for the future of public health.

The duration of COVID-19 and its variants, the recent outbreak of monkeypox, plus the highly pathogenic avian influenza, highlight the necessity for strong leadership from DrPH practitioners in preventing and responding to emerging diseases, along with PhDs in research and academia. The lack of strong public health guidance in the coordination and communication about COVID-19 and the vaccines underscored the need for effective public health leaders. It is time for public health schools and programs to begin offering online doctoral degrees that are accessible for learners who are not located near these schools and make it more convenient for working individuals, so future public health leaders are prepared.

Of the MPH survey respondents, over 10% had not heard of a DrPH degree and nearly one third did not know the difference between a PhD and DrPH. One program interviewee said that each year, she meets with department heads at her school to describe the DrPH degree. Oftentimes, the department heads do not know the difference between the two degrees. The DrPH Coalition^1^ is a small volunteer organization for DrPH degree holders. Its website clearly describes the differences between DrPH and PhD in terms of program requirements, program logistics, curriculum focus and the final project dissertation/thesis. DrPH programs should promote the degree, so that at least MPH degree holders are aware of the potential career path as practitioners. Ideally, a strong association for DrPH programs could inform institutions, as well as learners, about the difference between a PhD and a DrPH, so the latter is more recognizable.

When asked what has prevented the MPH survey respondent from going back to school, the majority of respondents cited the cost. Only half of the CEPH-accredited online public health doctoral programs offer any financial support for learners. Although tuition reimbursement is available at companies, over 15% of MPH respondents did not know if their employer offered repayment plans. To meet the public health challenges that lie ahead, programs need to provide financial support for future leaders, so they can enroll in programs that do not result in burdensome educational debt.

After cost, the next most cited reason that has prevented MPH degree holders from going back to school was time. One of the biggest advantages of online learning is time management (10). Courses for working students are often at the end of the day, so the class does not interfere with the workday. There is no commute time and time spent walking to the classroom, which may be across campus from parking or public transportation.

Those potential learners, who live long distances from public health programs and may be unable to quit their jobs and move near those programs, face inequitable challenges that their colleagues, who live near public health schools, do not encounter. Public health doctoral programs should train people where they live, not expect them to leave their current positions and move near the programs that would require moving expenses and a new job. Newly minted DrPH degree holders may remain near their resident programs after their training and not move back to areas where they may be needed.

A 2020 survey indicated that 52% of United States college students preferred online learning to in-classroom learning (11). Since the majority of students prefer online learning, programs should adapt to the users’ preferences by offering the desired modality.

Public health schools can follow the lead of UNC which re-engineered an in-person program with similar courses and the same faculty. As Babich mentioned, cohorts need to be small for the experiential learning. Instead, schools can mimic Johns Hopkins which has increased the number of cohorts, not the number of people within a cohort.

### Limitations

Unfortunately, the study did not include financial data on expenses, income, profitability, etc. Except for Babich, no one was willing to share their financial information, which would have been helpful for anyone contemplating launching an online public health doctoral program. Another limitation was the margin of error, which was increased to 8% from 5% since the actual sample size for MPH degree holders was lower than expected.

## Conclusion

To the best of our knowledge, no study with primary and secondary research has compared the features of CEPH-accredited online Doctor of Public Health programs or examined the interest in these programs by MPH degree holders. Who will keep the public healthy? MPH degree holders are waiting in the wings with their answer. They await the programs that will be accessible, efficient and equitable for them. Now is the clarion call for schools and programs in public health to provide online Doctor of Public Health degree programs for learners to become leaders and practitioners to keep the public healthy during the unknown public health challenges that lie ahead from epidemic to pandemic.

## Data availability statement

The original contributions presented in the study are included in the article/supplementary material, further inquiries can be directed to the corresponding author.

## Ethics statement

Ethical review and approval was not required for the study of human participants in accordance with the local legislation and institutional requirements. Written informed consent from the participants was not required to participate in this study in accordance with the national legislation and the institutional requirements.

## Author contributions

All authors listed have made a substantial, direct, and intellectual contribution to the work and approved it for publication.

## Conflict of interest

The authors declare that the research was conducted in the absence of any commercial or financial relationships that could be construed as a potential conflict of interest.

## Publisher’s note

All claims expressed in this article are solely those of the authors and do not necessarily represent those of their affiliated organizations, or those of the publisher, the editors and the reviewers. Any product that may be evaluated in this article, or claim that may be made by its manufacturer, is not guaranteed or endorsed by the publisher.

## Footnotes

^1^ drphcoalition.org
